# The Kynurenine Pathway and Kynurenine 3-Monooxygenase Inhibitors

**DOI:** 10.3390/molecules27010273

**Published:** 2022-01-02

**Authors:** Tamera D. Hughes, Osman F. Güner, Emma Carine Iradukunda, Robert S. Phillips, J. Phillip Bowen

**Affiliations:** 1 Department of Pharmaceutical Sciences, College of Pharmacy, Mercer University, 3001 Mercer University Drive, Atlanta, GA 30341, USA; tdhughes@email.unc.edu; 2 Department of Chemistry and Physics, Santa Rosa Junior College, Santa Rosa, CA 95401, USA; oguner@santarosa.edu; 3 Department of Chemistry, University of Georgia, Athens, GA 30602, USA; emmacarin.iradukunda25@uga.edu (E.C.I.); plp@uga.edu (R.S.P.); 4 Department of Biochemistry and Molecular Biology, University of Georgia, Athens, GA 30602, USA

**Keywords:** neurodegenerative diseases, Alzheimer’s disease (AD), kynurenine 3-monooxygenase (KMO), pharmacophore modeling, KMO inhibitors, kynurenine pathway (KP), neuroprotection, kynurenine, kynurenic acid, molecular dynamics simulation

## Abstract

Under normal physiological conditions, the kynurenine pathway (KP) plays a critical role in generating cellular energy and catabolizing tryptophan. Under inflammatory conditions, however, there is an upregulation of the KP enzymes, particularly kynurenine 3-monooxygenase (KMO). KMO has garnered much attention due to its production of toxic metabolites that have been implicated in many diseases and disorders. With many of these illnesses having an inadequate or modest treatment, there exists a need to develop KMO inhibitors that reduce the production of these toxic metabolites. Though prior efforts to find an appropriate KMO inhibitor were unpromising, the development of a KMO crystal structure has provided the opportunity for a rational structure-based design in the development of inhibitors. Therefore, the purpose of this review is to describe the kynurenine pathway, the kynurenine 3-monooxygenase enzyme, and KMO inhibitors and their potential candidacy for clinical use.

## 1. The Kynurenine Pathway

**Figure 1:**
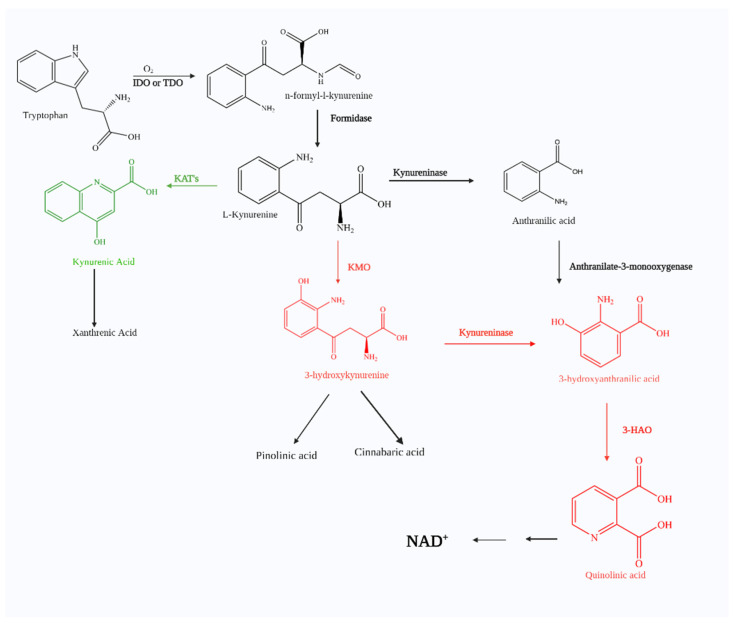
The kynurenine pathway. The kynurenine pathway is the essential metabolic pathway for the catabolism of tryptophan. There have been significant advances in researching the roles this pathway plays in neurodegenerative diseases. It is believed that inhibiting KMO would lead to a decrease production of toxic products downstream in this pathway, which would avoid the buildup of toxic metabolites that lead to certain disease. Kynurenine pathway metabolites—toxic metabolites in red, neuroprotective metabolites in green.

Tryptophan (TRP), an essential amino acid, can undergo catabolism through several different pathways. When metabolized by the serotonin (5-hydroxyptamine, 5-HT) pathway, 5-hydroxytryptamine or serotonin, a key hormone, affects mood, sleep, and pain [1]. Five gut-associated pathways, Actinobacteria, Firmicutes, Bacteroidetes, Proteobacteria, and Fusobacteria, have also proven to metabolize microbial tryptophan to produce neuro-active metabolites [2]. One pathway gaining particular interest is the kynurenine pathway (KP). The KP is the major mechanism for tryptophan catabolism with up to 99% of tryptophan being metabolized this way [1]. (Figure 1). Numerous pathological conditions involve KP, including neurological disorders (e.g., schizophrenia, depression, and anxiety), autoimmune diseases (e.g., multiple sclerosis and rheumatoid arthritis), peripheral conditions (e.g., cardiovascular disease and acute pancreatitis), and neurodegenerative illnesses (e.g., Huntington’s disease, Alzheimer’s disease, and Parkinson’s disease) and HIV. With KP implicated in a wide range of disease states, extensive effort has been invested into establishing connections and investigating potential therapeutic mechanism-based interventions.

The first step involves TRP oxidation by either indoleamine 2, 3-dioxygenase (IDO) or tryptophan 2, 3-dioxygenase (TDO) catalysis. The former has been found throughout the body; the latter is primarily expressed through the liver but has been found in the brain. Nevertheless, TRP oxygenation is catalyzed by either IDO or TDO to yield N-formyl-L-kynurenine. The next step involves the rapid conversion of N-formyl-L-kynurenine to L-kynurenine (KYN), which is the key and first stable intermediate of the KP by a formamidase. There are three possible metabolic fates for KYN, which involve biotransformations with (1) kynurenine aminotransferase (KAT) to form kynurenic acid (KynA), (2) kynureninase to form anthranilic acid, and (3) kynurenine 3-monooxygenase (KMO) to form 3- hydroxykynurnine (3-HK).

KynA is a known neuroprotective agent which has been attributed to nicotinic acetylcholine receptor (nAChR) binding as well as antagonistic effects through NMDA, AMPA, and kainite receptor binding [2,3,4]. In the presence of the KMO enzyme, KYN produces 3-hydroxykynurnine (3-HK) which further leads to the formation of picolinic acid, 3- HANA, cinnabarinic acid, and quinolinic acid (QUIN). Three metabolites, QUIN, 3-HK, and 3-HANA, have been shown to be neurotoxic. QUIN is an excitotoxin and N-methyl D-aspartate (NMDA) receptor agonist [3], whereas 3-HK and 3-HANA are involved with free radical formation [5].

### 1.1. Kynurenic Acid

One research stratagem has focused on the inhibition of key enzymes in the KP to shunt it towards a neuroprotective state. This idea is based on the assumption that kynurenic acid (KYNA) has neuroprotective abilities. Studies dating back as early as 1853 showed KYNA is produced in mammals; however, it was not until 1988 when it was shown to be present in the brain at concentrations of nanomolar quantities [3]. KYNA serves as a neuroprotective agent due to its antagonistic effects at the glutamate receptor and all three subtypes of ionotropic receptors, N-methyl-D-aspartate (NMDA), kainate, and alpha-amino-3-hydroxy-5- methyl-4-isoxazole propionic acid (AMPA) [4]. At high concentrations, KYNA binds to the glutamate site of the NMDA and AMPA receptors. Due to its high affinity for the glycine modulatory site of the NMDA in comparison to the glutamate, only low concentrations of KYNA are required for the glycine binding site. KYNA also serves as a neuromodulator in the nicotinic cholinergic system, increasing α4β2 nAChR expression and, more specifically, by inhibiting α7-nicotinic acetylcholine receptors (nAChRs) noncompetitively and voltage independently [5]. Though effects mediated by this process remain to be investigated, studies have shown that KYNA selectively binds to a G-protein-coupled receptor, GPR35, leading to its activation.

Five years after discovering the role kynurenic acid plays in epilepsy, another neuroprotective property was revealed—its ability to reduce ischemic brain damage [6]. Numerous studies have been published noting the effects of KYNA on neuronal activity and that decreased KYNA levels have been associated with major depression, Alzheimer’s disease, Huntington’s Disease, Parkinson’s Disease [7,8,9,10], etc. The regulation of KYNA has not only been implicated in neuronal diseases, but decreased levels of KYNA have also been implicated in diseases such as hypertension [11] and renal and colon cancers [12,13]. In addition to its activity on receptors, KYNA also has antioxidant properties, as it can scavenge hydroxyl, superoxide anion, and other free radicals [14].

Sometimes, too much of a good thing can have deleterious consequences, and one would be remiss to not disclose a few cases where elevated KYNA levels have led to disease. The earliest observations of KYNA date back to 1991. Here, it was shown that patients with schizophrenia presented with elevated kynurenic acid levels in the cerebral spinal fluid [15]. This discovery provided new insights in that KYNA also has possible effects on the glutamatergic and dopaminergic systems and could play a potential role in the pathogenesis of schizophrenia. A decade later, scientists confirmed this hypothesis. Not only do schizophrenic patients show elevated levels of kynurenic acid, but, also, these elevated levels of endogenous kynurenic acid increase the firing activity of midbrain dopamine neurons. This increase alters the effects of both nicotine and clozapine, leading to inhibitory responses of the ventral tegmental area (VTA) dopamine neurons that cause disrupted prepulse inhibition, an effect restored by antipsychotics [16]. Elevated levels of KYNA have also been implicated in rapid progression among lung cancer patients [17], HIV-related illnesses [18], cataracts [19], tick-borne encephalitis [20], and partial seizures in epileptic patients [19]. Most recently, KYNA has also been associated with antidepressant-like and antimigraine-like effects as well [21,22].

It would appear that there is a need to maintain a balance of KYNA; however, it has been presumed that the elevated levels of KYNA associated with these diseases are a result of the body’s failed attempt at protecting cells. All in all, kynurenic acid appears to be a metabolite of the kynurenine pathway that warrants further investigation in disease prevention.

### 1.2. Picolinic Acid and Other Possible Neuroprotective Metabolites

**Figure 2:**
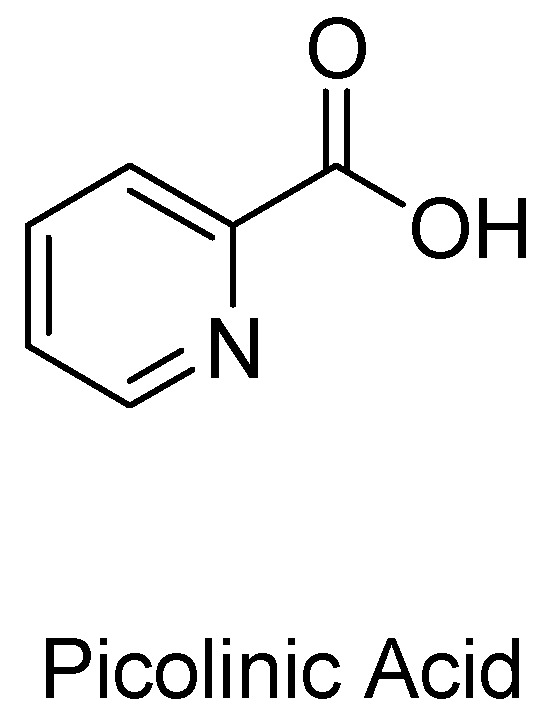
Structure of picolinic acid.

Picolinic acid (PIC) is another endogenous neuroprotectant metabolite of the KP pathway (Figure 2). Most of the research conducted on this monocarboxylic acid boasts its physical characteristics as an efficient chelating agent on a range of metals, including nickel, zinc, iron, cadmium, lead, and copper [23]. In fact, PIC metal complexes are now widely used as a means to introduce bioactive metals as dietary supplements in the body [24].

In addition to its physical characteristics, its physiological role has been shown to control cellular growth and have antitumor [25], antifungal [26], and antiviral [27] activities. Regarding its potential treatments involving the KP, PIC acts similarly to kynurenic acid, blocking quinolinic acid-induced neurotoxicity, but not the neuroexcitatory component [28]. While their effects seem similar, PIC is less potent than KYNA and has been suggested to work via a different mechanism—chelating endogenous zinc and/or attenuating calcium-dependent glutamate release [29,30].

**Figure 3:**
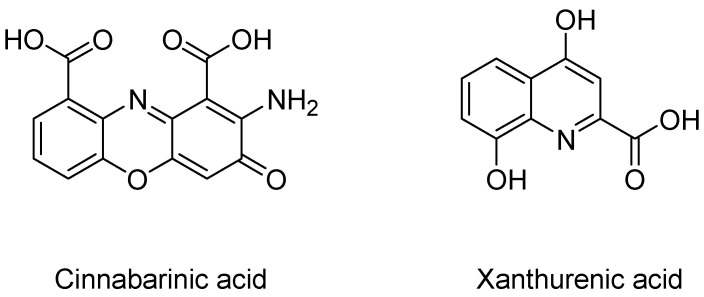
Structure of cinnabarinic acid and xanthurenic acid.

Cinnabarinic acid and xanthurenic acid, though less studied, are also thought to be neuroprotective metabolites of the kynurenine pathway. Structurally, cinnabarinic acid meets the requirements to interact with glutamate receptors, and xanthurenic acid (XA) appears to be a close analog of KYNA (Figure 3).

Recently it was discovered through the use of a high-performance liquid chromatography-tandem mass spectrometry analysis that cinnabarinic acid does indeed act as an endogenous orthosteric agonist of mGlu4 receptors, endowing it with neuroprotective activity [31]. In vivo and in vitro studies have also shown that the effects produced by XA appear to be mediated by the activation of mGlu2 and mGlu3 receptors [32]. Growing interests and studies on these two metabolites have shown that their activities may extend beyond the regulation of glutamate receptors; however, their involvement in the KP may reveal novel aspects in the complex interaction between the tryptophan metabolism and brain function.

### 1.3. 3-Hydroxykynurenine

Dissimilar to the other neurotoxic metabolites of the kynurenine pathway, the toxic effects of 3-hydroxykynurenine (3-HK) are independent of the NMDA receptor and solely result from the production of free radicals. 3-HK is mostly known for its ability to filter UV light in the human lens and its involvement in cataract formation [33]. 3-HK is a controversial metabolite, while mostly considered neurotoxic, it is also able to act as a scavenger and is involved in immunoregulation. For theories suggesting 3-HK is a powerful antioxidant, it must be noted that many studies observed a phenomena of concentration ranges outside those found in normal, healthy cells [34]. Even under physiological conditions, 3HK has been shown to produce reactive autoxidation products whilst also generating cytotoxic hydrogen peroxide and hydroxyl radicals [35].

**Figure 4:**
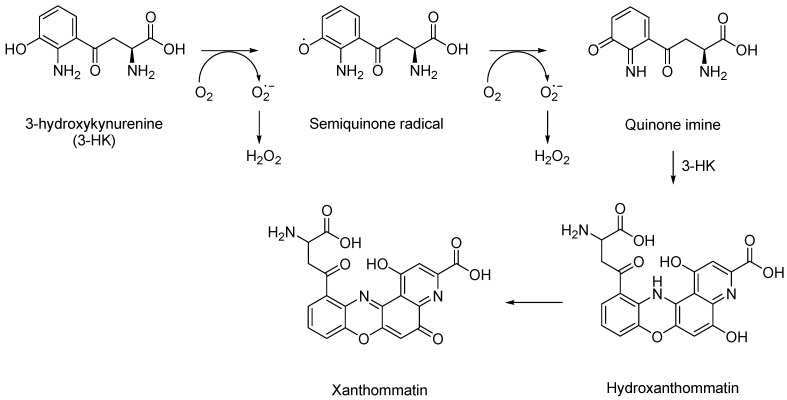
Oxidative reactions of 3-HK.

In general, the presence of an o-aminophenol moiety in 3-HK subjects it to several steps of oxidation reactions, precipitated by their oxidative conversion to quinone imines, which are accompanied by the reactive oxygen species (ROS) production of superoxide (O_2_^−^) and hydrogen peroxide (H_2_O_2_) [36] (Figure 4).

The cytotoxic action of 3-HK suggests a direct role for hydrogen peroxide (H_2_O_2_) and metal ions resulting in cell lysis from the intracellular accumulation of toxic levels of H_2_O_2_. Low concentrations of 3-HK (1–10 µM) are sufficient to produce ROS and induce cell death [37]. Concentrations greater than 100 micromolar of 3-HK have also been associated with toxicity to more than 85% of the cells in neuronal hybrid cell lines over a period of 24 h [38].

Abnormal levels of 3-hydroxykynurenine have been associated with the following: cardiovascular disease among patients with end-stage renal disease [39]; chronic renal insufficiency [40]; Alzheimer’s disease [41]; Huntington’s disease [42]; HIV-associated dementia [42]; vitamin B6 deficiency [43]. 3-HK also contributes to the production of quinolinic acid (QUIN), potentiating cellular toxicity mediated by QUIN formation. All in all, 3-hydroxykynurenine demonstrates significant cellular toxicity.

### 1.4. 3-Hydroxyanthranilic Acid

The product derived from the hydrolysis of 3-HK or the oxidation of anthranilic acid is 3-hydroxyanthranilic acid (3-HANA). Similar to 3-HK, 3-HANA has also been shown to play a role in the regulation of the immune system and is believed to scavenge NO radicals. 3-HANA, however, is prone to autoxidation and, having similar effects to its predecessor 3-HK, is mostly associated with toxicity and the formation of superoxide anions, which eventually leads to the damage of proteins. In fact, because 3-HANA rapidly undergoes autoxidation, the deleterious effects of 3-HK are actually due to its metabolite 3-HANA [44]. Neuronal cell cultures exposed for long periods and high concentrations of both 3-HK and 3-HANA were specifically characterized to present the toxic pro-oxidative effects of these metabolites [45]. 3-HANA, such as several o-aminophenolic compounds, may also act as an endogenous carcinogen. The role of abnormal tryptophan metabolism has been proven to contribute to the etiology of human bladder cancers; elevated levels of these metabolites, including 3-HANA, are believed to cause spontaneous bladder tumors [46].

The neurotoxic metabolite 3-HANA, along with quinolinic acid, which was be discussed below, have also been studied in several animal studies, and an increase in these metabolites has been reported in epileptic mice [47]. Additionally, during these animal studies, it was observed that the increase in the toxic metabolites is found together with increased levels of kynurenic acid, which led to the idea that KynA also contributes to the epileptic state. KYNA levels were increased in response to combat these epileptic mediators, and this resulted in the conclusion that elevated levels of kynurenic acid induce sedation and suppress convulsions, suggesting a functional antagonism of the excitatory amino acid receptors [48].

### 1.5. Quinolinic Acid

Studies elucidating the toxic effects associated with quinolinic acid (QUIN) have been more conclusive and discovered QUIN to be notorious for cytotoxicity and neurotoxicity. In healthy tissue, the concentration of QUIN present in the brain is low compared to blood and systemic tissues. An immune response, however, shifts levels of QUIN to rise dramatically [49]. Macrophages, microglia, and dendritic cells are the major generators of QUIN under inflammatory conditions. Astrocytes and neurons are capable of taking up and catabolizing QUIN. In this case, however, the catabolic system is easily saturated, further resulting in the toxic accumulation of QUIN within the cells [50].

For the past two decades, the destruction caused by QUIN has been attributed to its ability to activate the neuronal NMDA subtype of glutamate receptors. While this remains true, additional mechanisms have also been shown to contribute to this complex neurotoxicity. Dangerously enough, QUIN is not only capable of potentiating its own toxicity, but also other excitotoxins such as glutamate, whilst inhibiting the reuptake of glutamate by astrocytes. QUIN compromises the integrity of the blood–brain barrier (BBB), generates reactive oxygen intermediates, and depletes endogenous antioxidants and peroxidation of lipid molecules [51]. Even with a basic understanding of quinolinic acid, most are familiar with the above mechanisms of QUIN toxicity. For this reason, newer studies were presented to expose mechanisms that have been identified in recent years.

An increased production of nitric oxide has been shown in rodents and human neurons and astrocytes following the induction of neuronal nitric oxide synthase by QUIN [52]. The dysregulation of the astroglial function and gliotoxicity is also a novel theory to add to QUIN’s ability to kill neurons, redefining the cellular connection between neurons and glia in both physiological processes and pathological conditions [53]. QUIN increases the phosphorylation of cellular structural proteins, which damages the cytoskeleton of neurons and astrocytes [54]. This destruction of the cellular structure has brought significant interest to QUIN’s role in hyperphosphorylated tau seen in Alzheimer’s disease (AD) [55]. Several studies have confirmed the pathological role QUIN plays in the development of many diseases. Elevated concentrations of QUIN have proven to contribute directly to HD, AD, AIDs-related dementia [50], poliovirus brain infection [56], multiple sclerosis [57], cerebral ischemia [58], cerebral malaria [59], and epilepsy [60].

In summary, QUIN is a potent toxic metabolite implicated in many disease states. For this reason, therapeutic strategies are necessary to diminish the effects of QUIN. The goal is to shift the balance of the kynurenine metabolites through the inhibition of KMO to favor KynA, which, as stated above can counteract the effect of quinolinic acid.

### 1.6. Kynurenine 3-Monooxygenase (KMO)

Of greatest importance in the kynurenine pathway is the enzyme KMO. The remaining chapters of the literature review focus in depth on this compelling enzyme. KMO is a flavindependent hydroxylase that catalyzes the hydroxylation of L-kynurenine (L-Kyn) to 3-hydroxykynurenine (3-HK) in the kynurenine pathway (KP) (Equation (1))

(1) $$L-\mathrm{kynurenine}+\mathrm{NADPH}+O_{2}\;\overset{\mathrm{KMO}}{\to }\;3-\mathrm{hydroxykynurenine}+{\mathrm{NADP}}^{+}+{\;H}_{2}O$$

KMO belongs to a family of NAD(P)H-dependent flavin monooxygenase (FMO) [61]. It is encoded by one gene, has an FAD cofactor, utilizes either NADPH or NADH, releases NADP^+^ /NAD^+^ after flavin reduction, and has one dinucleotide-binding domain, which simply categorizes it as a Class A flavoprotein aromatic hydroxylase [62,63]. There are 437–513 residues that make up all KMO proteins, with human KMO being 486 amino acids in length and weighing approximately 50 kDa [64,65]. KMO is responsible for the conversion of L-Kyn to neurotoxins 3-HK, 3-HANA, and QUIN. Due to its involvement in a number of diseases, this enzyme has been purified and studied from several sources.

## 2. KMO in Disease

Under normal physiological conditions, cytokines, growth factors (GF), and hormones regulate the levels of kynurenine pathway metabolites. Under inflammatory conditions, however, there is an upregulation of the KP enzymes, particularly KMO, that increase the production of toxic KP metabolites in the brain. The dysregulation of the kynurenine pathway and increased levels of toxic metabolites have been implicated in various disease states, including neurological disorders such as Huntington’s disease (HD), Alzheimer’s disease (AD), Parkinson’s disease (PD), and Amyotrophic lateral sclerosis (ALS) epilepsy; affective disorders schizophrenia, depression, and anxiety; autoimmune related diseases rheumatoid arthritis (RA), multiple sclerosis (MS), and HIV-related dementia; peripheral conditions such as cardiovascular disease and ischemic stroke, and malignancies such as hematological neoplasia and colorectal cancer. The inhibition of KMO became a potential therapeutic strategy to rebalance the KP in hopes of mitigating and/or preventing disease progression since it sits at the key branching point of the KP. Inhibiting KMO will not only decrease the levels of toxic metabolites 3-HK and QUIN, but also increase the levels of the neuroprotective KYNA available for metabolism by kynurenine aminotransferase.

### 2.1. Huntington’s Disease

When rodents were injected intrastriatally with excitotoxin QUIN, their symptoms were observed to be similar to that of the cardinal features of Huntington’s disease [66]. In fact, HD was the first disease found to result from KP dysfunction. HD is a dominantly inherited genetic disorder characterized by neuronal loss in the striatum leading to the gradual onset of motor incoordination and cognitive decline [67].

Genetically, HD is the result of a mutant Huntingtin gene (mHTT) [68]. The Huntingtin gene (HTT or IT15 “interesting transcript 15”) encodes for the Huntingtin protein. While the function of HTT is unclear, the huntingtin gene is ubiquitously expressed and is essential for normal development (“HTT huntingtin [Homo sapiens (human)]—Gene—NCBI,” n.d.). Studies have shown that QUIN administration induces HTT in rats [69]. QUIN-administered rodents are the most reliable animal models to study HD pathogenesis and potential therapies [70]. It is an accepted theory that the overstimulation of the N-methyl-D-aspartate (NMDA) receptor is pivotal in the role of HD [71]. As new experimental HD models become accepted, research has shifted towards a “kynurenergic hypothesis of HD,” forcing scientists to look further at the role the kynurenine pathways play in triggering the disease. It has been demonstrated that KYNA concentrations disproportionately regulate neuronal vulnerability to QUIN in the striatum, with mutant mkat-2 −/− mice showing a susceptibility to QUIN increasing with decreasing KYNA levels [72]. While brain tissue retrieved from postmortem patients with HD shows no increase in QUIN, suggesting the QUIN level elevation to be absent in the later stages of HDl; in the early stages of the disease, there are significantly increased levels of both QUIN and 3-HK in the striatum and frontal cortex [73,74]. Moreover, 3-HK levels were substantially higher in brain tissues that were taken posthumously from HD and AD patients [75]. Animal studies using HD mouse models have since verified the increase in QUIN and 3-HK concentrations observed in human studies [42]. Inevitably, an imbalance among the neurotoxic and neuroprotective metabolites from the KP contribute to HD pathology [76]. Currently, there is no cure for HD, and the few drugs indicated to treat the disease only provide symptomatic relief. To control the hallmark symptom of HD (chorea), tetrabenazine, neuroleptics, and benzodiazepines are used [77,78]. Psychotropic drugs help to alleviate psychiatric disturbances and anti-Parkinson’s agents assist to mitigate hypokinesia and rigidity, but may induce chorea [78]. Despite treatments with these agents, HD patients ultimately succumb to life-altering complications resulting from worsening muscle coordination, declining cognitive function, and death. Now that research into the pathophysiology of HD has shifted towards a kynurenergic model, it is conceivable that an improved and sustainable treatment can arise from KMO inhibition.

### 2.2. Alzheimer’s Disease

Alzheimer’s disease (AD) is a neurodegenerative disorder that causes a progressive decline in cognition and memory, and, ultimately, it results in dementia and death. The incidence of the disease increases with age, and it has been estimated that AD may affect more than 40% of the population over the age of 84. Although drugs have been developed to treat the cognitive symptoms of AD, there are no therapies presently available that can stop or reverse the progression of the disease. It has been an accepted dogma in the field that the cognitive decline in AD is a result of the presence of unusual structures in the brain, called plaques, which are composed of an aggregated protein called amyloid. It has been shown that amyloid is produced by the processing of a precursor protein molecule to produce a number of peptides containing 37–43 amino acids, known as Aβs, which associate to form insoluble structures called fibrils. The accumulation of insoluble Aβ in cells was thought to induce apoptosis, or programmed cell death, in the affected neurons. The molecular mechanism by which Aβ plaques induce neuronal cell death is still unclear. In fact, recent studies suggest that Aβ plaques may not be the cause of neurodegeneration in AD, but the result [79].

Until now, pharmacologic treatments for AD have tried to control cognitive deficits through the administration of acetylcholinesterase inhibitors in an effort to increase acetylcholine levels in the CNS, but such strategies have only had modest success [80]. Interestingly enough, Donepezil (Aricept), one of the drugs currently used to treat Alzheimer’s disease, was developed using molecular modeling [81]. Newer drugs have been introduced that target plaques (Aβ aggregation inhibitors) or target proteases such as β- and γ-secretase (Aβ formation inhibitors). Recent reports from several clinical trials have indicated that the efficacies of the drugs are disappointingly low [82]. Hence, there is a pressing need for the discovery of novel AD therapeutics to enter the development pipeline that are focused on alternative targets. These new drug entities will target the kynurenine pathway of the tryptophan metabolism in humans, specifically kynurenine monooxygenase (KMO).

Recent studies have shown that one of the Aβ peptides, Aβ (1–42), is a potent and specific inducer of the kynurenine pathway of the tryptophan metabolism in neurons [82]. Two of these tryptophan metabolites, 3-hydroxykynurenine (3HK) and 3-hydroxyanthranilic acid (3-HANA), are known and are usually generically called kynurenines. QUIN has been found in high concentration in AD plaques [55]. Furthermore, QUIN has also been shown to induce the phosphorylation of tau protein in human neurons, and hyper-phosphorylated tau has been linked to AD. Thus, it appears that the etiology of AD may be related, at least in part, to the formation of abnormally high levels of neurotoxic kynurenines and/or QUIN as a result of the overexpression of the kynurenine pathway by neuro-inflammation. Whether directly causative of AD or not, high levels of these toxic compounds are unquestionably undesirable. This hypothesis is supported by a recent publication, showing that an inhibitor of kynurenine monooxygenase attenuates the development of neurodegeneration in Alzheimer’s mice, even if it does not cross the blood–brain barrier [83]. Though tryptophan, KYN, and 3HK can cross the BBB, it is important to understand that the KMO enzyme is not solely expressed in the brain, but is highly expressed in the liver, kidney, endothelial cells, and monocytes. Blocking KMO in the periphery deters the KP from its toxic metabolites and pushes it towards the formation of KYNA, a protective metabolite. Thus, enzymes in the kynurenine pathway are potentially novel therapeutic targets for the treatment of AD that have not been significantly explored.

### 2.3. Parkinson’s Disease

Closely following Alzheimer’s disease is Parkinson’s disease, the second most often occurring neurodegenerative disease. Characterized by disorders of the motor system resulting from the loss of dopamine-producing brain cells, at least one million individuals in the United States alone are suffering from Parkinson’s disease (PD) [84]. The etiology behind Parkinson’s disease is unknown, but genetic as well as aging and environmental factors are believed to interplay in the genesis of the disease. Nevertheless, a key histopathologic feature has been shown to result from the disease—the degeneration of dopaminergic neurons in the substantia nigra that project to the striatum [85]. Neuro-inflammation is also shown to occur in conjunction with dopaminergic neuron destruction even before PD symptoms begin. In response to this neuro-inflammation, microglial cells become activated to release a host of inflammatory mediators, which further perpetuate neuro-inflammation and neurotoxicity. As mentioned above, the kynurenine pathway has been implicated in many disorders involving neuro-inflammation and neurotoxicity, proving to be a major regulator of the immune response. Reasonably enough, scientists began searching for links between the KP and PD. In 1992, scientists discovered that PD brain tissues not only revealed decreased levels of dopamine (DA), but also uncovered significantly decreased concentration levels of kynurenine and kynurenic acid, as well as increased levels of 3-HK, regardless of whether or not the patient was receiving treatment with L-DOPA [10]. The conclusions drawn from this discovery were that the nicotinamide-adenine dinucleotide (NADH) produced via the KP could be responsible for NADH:ubiquinone oxidoreductase (complex I) present in dysfunctional PD mitochondria. In fact, nearly all of the neuroactive compounds produced via the KP can be demonstrated throughout PD. Recall that KP also produces QUIN, the precursor of NADH and an agonist of the NMDA receptor, which can be found on dopaminergic neurons [52]. Therefore, not only do those activated microglial cells release a host of inflammatory mediators, but one of those mediators, QUIN, is also produced by these cells and has been shown to co-localize with dopaminergic neurons in the substantia nigra of macaques treated with the MPTP, a neurotoxin causing parkinsonism [86]. 3-HK, a well-known toxin to striatal neuronal cultures, also induces oxidative damages in the putamen and substantia nigra pars compacta (SNpc), as it generates reactive oxygen species and initiates apoptosis [86,87]. Similar to most neurodegenerative diseases, currently, there is no treatment for PD; therapies only assist in mitigating the disease symptoms. Levodopa (L-dopa) is a mainstay treatment in PD, as it increases the concentrations of dopamine that are reduced in PD patients, and, such as many PD treatments, it does not arrest neurodegeneration and is only briefly effective, as long-term therapy has been associated with severe side-effects (“Drug Result Page—MICROMEDEX,” n.d.). These side effects, however, have been shown to subside following the administration of KMO inhibitors RoRo-61-8048 and nicotinyalanine without disrupting the effects L-dopa has on PD [86]. Thus, whether a therapeutic target for treating PD symptoms or at least diminishing side effects of current treatments, novel approaches must be explored in developing therapy for PD.

### 2.4. Epilepsy

Though not a neurodegenerative disease, epilepsy is a neurological disease characterized by disturbances in the electrical activity of the brain [88]. In layman’s terms, seizures describe epilepsy. Scientifically, epilepsy is defined as the occurrence of at least two unprovoked seizures separated by 24 h [89]. The fourth most common neurological disorder, following stroke, migraines, and AD, the World Health Organization (WHO) estimates at least 2.4 million people are being diagnosed with this disease each year, with more than 65 million people suffering worldwide [90]. Earlier in this chapter, it was discussed briefly that KYNA, QUIN, and 3-HANA concentrations were measured in epileptic mice. Increased levels of the neurotoxic metabolites, quinolinic acid and 3-HANA, were reported in these mice, and KYNA levels were increased in response to combat these epileptic mediators [47]. This resulted in the conclusion that elevated levels of kynurenic acid induce sedation and suppress convulsions, suggesting a functional antagonism of the excitatory amino acid receptors [48]. Again, for reiteration, QUIN stimulates the NMDA receptors, and excessive stimulation is essentially what results in the over excitation leading to epileptic seizures. Once it was discovered that KYNA had anticonvulsant properties and QUIN convulsant properties, it became arduous to deny the physiological role that KP plays in epilepsy. This led researchers to study the effects of the perturbation of the kynurenine pathway in epilepsy. Since then, publications have shown that KMO inhibitors produce elevated KYNA levels in the brain, blood, liver, and hippocampal regions in rats; thus, shifting the balance of the KP towards the neuroprotective KYNA, proving yet another disease that KMO inhibitors could therapeutically target.

### 2.5. Neuropsychiatric Disorders

Most of the work dealing with kynurenine metabolism focuses on how it relates to neurodegenerative diseases. The disruption of the KP, however, can also have considerable effects on behaviors relevant to neuropsychiatric indications, including schizophrenia, depression, and attention-deficit/hyperactivity disorder (ADHD). Neuro-inflammation has been reported in conjunction with psychiatric disorders to produce pro-inflammatory mediators that are capable of activating tryptophan catabolism via the kynurenine pathway and shifting it towards the neurotoxic arm where NMDA receptors are stimulated. The serum or plasma imbalance of KP metabolites is present in the cerebrospinal fluid (CSF), as well as certain areas of the brain. These metabolites interact with other neurochemicals, contributing to the pathophysiological mechanism in psychiatric disorders. For this reason, kynurenine metabolites can also be used as biomarkers for certain neuropsychiatric diseases [91]. Tryptophan is the precursor of serotonin, the neurotransmitter known for its overall effect of contributing to happiness and well-being [92]. The depletion of tryptophan results in the depletion of serotonin, yielding a depressed mood state. Depression, also referred to as clinical depression or major depressive disorder (MDD) [93,94], is a multifactorial mood disorder that is characterized by anhedonia [95]. It is well documented in patients with MDD that IL-4, IL-10, and other anti-inflammatory cytokines are unmatched in antagonizing pro-inflammatory cytokines IL-2, IL-6, soluble IL-6 receptor, TNF α and IFN- γ [91]. These pro-inflammatory mediators are capable of stimulating both IDO and KMO activity in the KP, not only depleting TRP, but also shifting the KYN metabolism towards 3-HK and QUIN production [96]. The microglia are commonly accepted as the place where the brain KMO activity increases in response to peripheral inflammation, but studies have also shown an increase in NeuN-positive neuronal cells in the hippocampal dentate gyrus [97]. For this reason, it has been proposed that the imbalance of neurotoxic metabolites and KYNA may be responsible for exposing the astrocyte–microglia-neuronal networks to environmental factors, such as stress, which may possibly be a contributing factor to the intermittent and persistent nature of MDD [91]. Studies suggest that depressed patients not only have a reduction in the availability of TRP, but also a higher activity of TRP breakdown and significantly decreased levels of plasma KYNA [7]. This trend is not only present in adult depression, but also in adolescent depression [98].

Another neuropsychiatric disorder in which immune activation has been observed is schizophrenia. Schizophrenia is one of the most recondite and challenging neuropsychiatric disorders, characterized by hallucinations, delusions, disorganized thinking and speech, grossly disorganized or abnormal motor behavior (including catatonia), and negative symptoms [99]. The hyperactivity of dopamine or the dopamine hypothesis has been accepted to describe the pathophysiology of schizophrenia. However, it only gives a partial analysis of what occurs in the schizophrenic brain, which has led scientists to extend the hypothesis to the dysregulation of neurotransmitters in the brain. Of particular interest is the view on the hypothesis of the glutamatergic hypofunction. The glutamatergic neurotransmitter system is the most widespread excitatory neurotransmitter system in the central nervous system, and shifts its function, either hypoactivity or hyperactivity, can result in toxic neuronal reactions [100]. Closely resembling the hypothesis of glutamatergic hypofunction, stands the KYNA hypothesis. Studies have shown that the brains and CSF of post mortem patients with schizophrenia contained elevated concentrations of KYNA, supporting this hypothesis, which believes an overproduction of kynurenic acid leads to the pathophysiology of the disorder [101]. Symptoms seen in schizophrenia have also been reproducible via the administration of KYNA, concluding that KYNA is a biologically important modulator of glutamatergic neurotransmission within the brain [16]. These findings are simply unexplainable, as KYNA is usually shown to offer neuroprotection, and though the majority of TRP research on schizophrenia concentrates only on KYNA, studies suggest an upregulation of TRP metabolism with an inflammatory background also plays a role. It would appear that there is a need to maintain a balance of KYNA; it has been assumed, however, that the elevated levels of KYNA that lead to disease are a result of the body’s failed attempt at protecting cells.

### 2.6. Stroke

Cardiovascular disorders (CVDs) and their consequences account for one out of every three deaths in the United States [102]. CVDs are the most serious health problem of the Western world, with over 800,000 people in the United States having died from heart disease, stroke, and other cardiovascular diseases in 2013. As defined by the American Heart Association (AHA), CVD refers to conditions that involve narrowed or blocked blood vessels leading to myocardial infarctions, arrhythmias, heart valve problems, and/or strokes. When these narrowed or blocked blood vessels reduce the flow of oxygen and nutrients to brain tissues, the brain cells begin to die. This is considered an ischemic stroke. Stroke is the second cause of death in individuals older than 60 and the fifth leading cause of death in individuals aged 15–59 [103]. Many stroke survivors are left with chronic, debilitating health problems requiring consistent dependency on caregivers and a continuous recovery and rehabilitation regimen. While numerous drugs are used for stroke prevention, only a few therapeutic options are available for stroke treatment. The recombinant tissue plasminogen activator (rtPa) has been the mainstay treatment for acute ischemic stroke (AIS); however, only 2–5% of stroke patients receive rtPa treatment due to its strict eligibility criteria and a narrow therapeutic window [103]. Over the last few decades, convincing evidence has been generated, stating that oxidative stress is one of the most potent inductors of endothelial dysfunction and is involved at all stages of atherosclerotic plaque evolution [104,105]. This evidence led researchers to study the KP as it relates to ischemic stroke. Barone and Feuerstein were one of the first to theorize that anti-inflammatory responses with increased signs of oxidative stress rapidly follow a stroke [106]. In 2010, the role of the tryptophan metabolism along the kynurenine pathway in AIS was discovered. Not only did this study reveal that post stroke inflammation activated the KP, but also that the activity of this upregulation correlated with stroke severity and long-term stroke outcomes [107]. This change in the kynurenine metabolism is easily detectable, as it was soon discovered that kynurenines produced in response to cerebral impairment can easily be found in the periphery, suggesting the accessibility of kynurenines beyond the BBB [108]. Recall the KMO inhibitor (*m*-nitrobenzoyl)-alanine (mNBA). Studies have also shown that not only does administering mNBA lead to neuroprotection in rats, but it also results in the reduction in ischemic neuronal damage introducing an additional therapeutic route for KMO inhibition—the reduction in neuronal loss in brain ischemia [6]. The overactivation of the KP has been implicated in numerous pathologies affecting the brain, and such is the case of stroke. Correlating the KP in response to AIS is a new research avenue, and further studies are required to determine the exact mechanism, which modulates the activation of KP after cerebral ischemia. Nevertheless, KMO inhibition has the potential to serve as a pharmacological tool in both acute and chronic phases of stroke.

### 2.7. Other Immune-Related Illnesses

**Figure 5:**
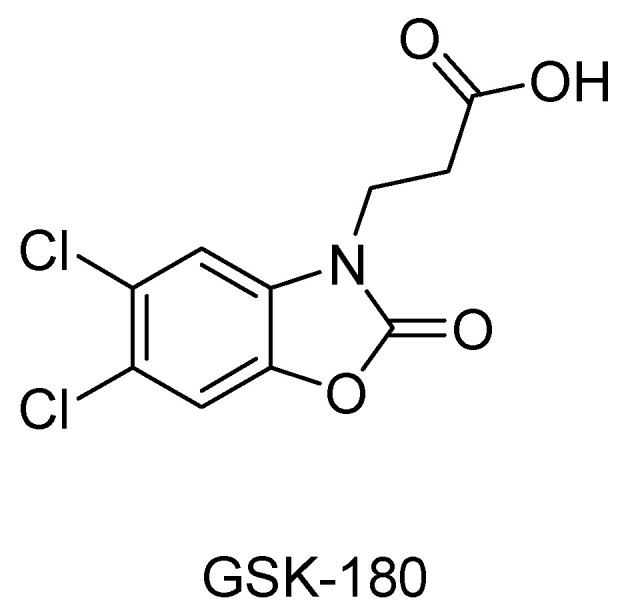
KMO inhibitor developed by GlaxoSmithKline; summary.

It would take multiple lifetimes to exhaust all the disorders resulting from the dysfunction of the kynurenine pathway. In order to complete this research, a few more studies discussing the kynurenine pathway, specifically KMO, in other disease states were reviewed. The upregulation of KMO currently serves as an independent prognostic biomarker to identify certain liver malignancies, particularly hepatocellular carcinoma (HCC). HCC patients who express increased KMO activity are known to have unfavorable clinical outcomes compared to those who do not [109]. The upregulation of KMO also plays a critical role in triple-negative breast cancer progression and metastasis [110]. The pharmacological inhibition of KMO with GSK180 granted therapeutic protection in acute pancreatitis rodent models by preventing multiple organ failure (Figure 5). These discoveries opened up a new strategy for drug discovery in critical illnesses [111].

## 3. KMO Mechanisms and Properties

The elucidation of the catalytic mechanism of KMO was made possible from studies on KMO from the bacteria *Pseudomonas fluorescens* [112]. In order to understand the catalytic mechanism, one must first explore the characteristics typical of class A flavoprotein aromatic hydroxylase monooxygenases, to which class the KMO enzyme belongs.

**Figure 6:**
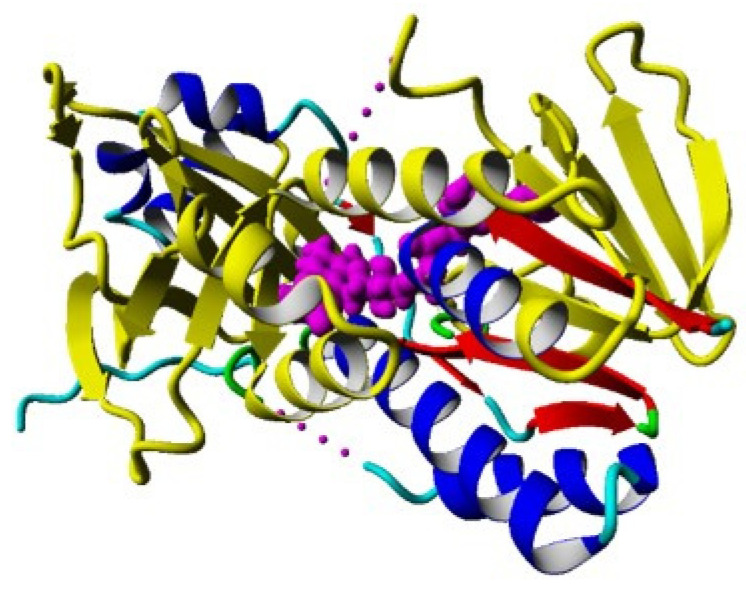
Beta–alpha folding in human KMO depicting Rossmann fold in yellow and FAD co-factor in magenta.

Flavoprotein monooxygenases are a diverse class of oxidative biocatalysts that have been divided into six categories (A–F). Class A monooxygenases are encoded by one gene, comprised of a flavin adenine dinucleotide (FAD) cofactor, use either NADPH or NADH to release NADP^+^ or NAD^+^ after flavin reduction, and have one dinucleotide binding domain binding to FAD represented as a Rossmann fold [63]. A Rossmann fold simply characterizes a secondary structure with an alternating motif of beta sheets and alpha helices, and is of importance because this domain non-covalently binds the FAD cofactor and also contains the active site of the enzyme for KMO (Figure 6).

Similar to many oxidoreductases, the catalytic cycle of KMO can be divided into two half reactions, a reductive half and an oxidative half. The binding of KYN to KMO is relatively slow, causing the reduction half of this reaction to be KYN dependent. Once kynurenine and NADPH bind to KMO and the FAD cofactor is reduced by NADPH, NADP^+^ disassociates from the enzyme. Once reduction occurs and NADP^+^ dissociates, the enzyme complex reacts with molecular oxygen to form a flavin hydroperoxided species that transfers an oxygen atom to KYN. The resultant hydroxy-flavin intermediate is rapidly dehydrated prior to product release. The now oxidized enzyme complex consequently experiences a conformational change that facilitates the release of the product 3-HK, making the release of 3-HK the rate-limiting step of this mechanism. As a result of this requirement, there is a change in the spectrum of the oxidized enzyme.

### 3.1. Efforts to Isolate and Characterize KMO

While KMO plays a key role in the kynurenine pathway of mammals, it has also been found in certain degradation pathways for some species of bacteria and fungi. The first partial purification of the KMO protein, then known as L-kynurenine hydroxylase, was obtained from rat liver mitochondria in 1957 [113]. Though this isolation occurred over six decades ago, mammalian KMO continued to be a difficult protein to isolate due to solubility issues (it was not until February 2018 that a crystal structure of human KMO was published). Nevertheless, some structural information was made available through sequence analyses, functional studies, and enzyme purification from several sources.

Tissue distribution studies showed mammalian KMO to be highly expressed in the liver and present in small amounts in the kidney. KMO has also been found in endothelial, macrophage, microglial, and monocytic cells. Yet, while expressed in a wide array of cell types, amazingly enough, very low levels of KMO have been found in brain cells [114].

### 3.2. Structures

Though crystal structures for KMO were not available until recently, insight into the structural information of KMO was made possible through sequence analyses and functional studies. The first crystal structure of KMO in 2013 of a truncated *Saccharomyces cerevisiae* enzyme was published in 2013PDB 4J36 and 4J33; [115]). The team described the enzyme not only in the free form, but also in complex with the tight-binding inhibitor UPF648, 2-(3, 4-dichlorobenzoyl-cyclopropane-1-carboxylic acid. A successful KMO inhibition could require small molecular compounds to cross the blood–brain barrier. Therefore, knowing the bioactive conformation of an enzyme-inhibitor would assist in the development of new inhibitors. Presumably, the differences between the human KMO and this X-ray structure were small enough to consider a structure-based drug design. Unfortunately, they were unable to crystallize the structure with kynurenine bound. Both structures were solved as a putative dimer with PDB 4J33 having a resolution of 1.82 Å and PDB 4J36 with a resolution of 2.13 Å. The truncated (truncated at residue 394) *S. cerevisiae* KMO in the absence of a ligand in the active site is currently the highest resolution the KMO structure has had published to date. The KMO fold, similar to other flavin-dependent hydroxylase structures, features a glutathione reductase (GR-2) Rossmann fold for flavin adenine dinucleotide (FAD) binding that interacts with a part of the domain holding five β-sheets and four α-helixes [116]. It was found that UPF648 binds closely to this domain and causes a conformational change, which denies KYN access to bind with the subsequent inhibition of KMO activity. Conserved residues Arg83 and Tyr97 bind the UPF-648 carboxylate, and conserved hydrophobic residues Leu221, Leu234, Met230, Ile232, Phe246, Phe322, and Pro321 flank the aromatic dichlorobenzene moiety. Mutagenesis and functional assays have found these residues are conserved across different organisms, allowing the translation of this work to human KMO. *S. cerevisiae* and human KMO, which share 38% identity and 51% similarity, further allowing the experimental structure of *S. cerevisiae* to be an irrefutable template for docking screens using virtual compound libraries and aiding in the development of novel inhibitor scaffolds.

### 3.3. Pseudomonas Fluorescens

In addition to the *S. cerevisiae* KMO crystal structure, a KMO crystal structure from *Pseudomonas fluorescens* has also been solved. The tryptophan catabolism via KMO has been identified in a number of bacteria, including *P. fluorescens*, *Cytophaga hutchinsonii*, and *Ralstonia metallidurans* [117]. The enzyme from *P. fluorescens* (PfKMO) has demonstrated adequate stability to be expressed heterologously and isolated. In 2007, it was cloned and expressed in *Escherichia coli* with a high yield of soluble protein and successfully purified using a process of streptomycin sulfate precipitation, ammonium sulfate fractionation and anion exchange chromatography [62]. The solved structures include no ligand in the active site, L-kynurenine bound, and other inhibitors bound. This study allowed for the first substantially thorough mechanistic illustration of KMO and firmly proved KMO to be associated with the flavoprotein aromatic hydroxylases (FAH) enzyme class.

While the substrate-absent model depicts a similar structure to that of the substrate-bound PfKMO, a significant conformational change presented in the position of the C-terminal domain. For this reason, it was concluded that the C-terminal domain must play an integral role in the binding of substrates [118]. When PfKMO does not hold a substrate, the enzyme is said to be in an “open” conformation. It is theorized that this “open” conformation allows for the accelerated binding of substrate and product release. Once a substrate binds to PfKMO, the C-terminal domain then moves towards a “closed” conformation observed in the KYN-bound structure. Understanding substrate binding could allow one to apply this knowledge to the development of effective KMO inhibitors.

### 3.4. Human KMO

Until recently, human KMO (hKMO) had not been crystallized. Complications with solubility posed a major challenge to researchers who were trying to isolate an active mammalian KMO enzyme. During this time, recombinant enzymes expressed in cell lines were used to perform most of the work with mammalian KMO. It was confirmed that the hKMO also contained one non-covalently bound FAD cofactor per monomer due to active hKMO expressed in human embryonic kidney cells (HEK293) and later in COS-1 cells [64,65]. COS-7 cells expressing pig KMO helped to further prove the essentiality of the C-terminal domain for activity and that the residues in the last twenty amino acids of this domain were necessary for localizing the enzyme in the outer mitochondrial membrane, which is thought to cause the insolubility of KMO [119].

Korean researchers reported the first structure of human KMO [120]. Their findings may be summarized as: the crystal structure was solved to a resolution of 2.1 Å after engineering the deletion mutant hKMO-374 (residues 1–374), in which the transmembrane domains or TMDs were deleted in order to obtain a human KMO protein suitable for crystallization. As described earlier by Hirai [119], the hKMO can be located in the mitochondrial outer membrane, contains two transmembrane domains (TMDs), and a C-terminal region responsible for mitochondrial signaling. A catalytic mechanism composed of two domains, again, typically found in FAH enzyme class, was revealed in hKMO-374. The first domain contained the FAD-binding region bound at full occupancy (1:1 stoichiometry), while the second contained the small N-terminal domain consisting of alpha helices and an antiparallel beta sheet. The findings from this study further provide insight into the KMO enzyme and could likely facilitate the development of KMO inhibitors.

### 3.5. KMO Inhibitors

**Figure 7:**
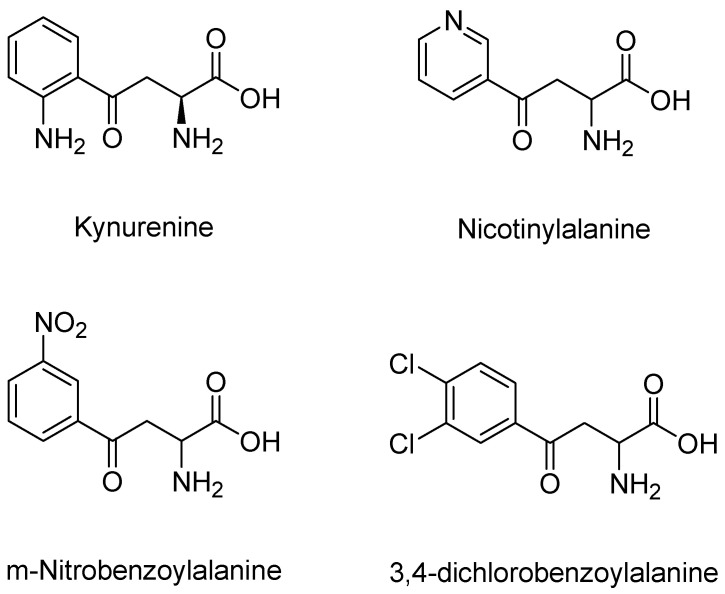
Analogues of endogenous ligand, kynurenine.

Developing KMO inhibitors for clinical use has proven to be difficult before the crystallization of KMO. The lack of structural information resulted in the early inhibitors of KMO being based on analogues of the endogenous ligand, L-kynurenine. Nicotinylalanine (Figure 7), structurally very similar to L-Kyn, demonstrated a weak nonspecific inhibition of both KMO and kynureninase, and when used in vivo, kynurenic acid levels increased in rat brain tissue [121,122]. A rational structure-based design followed the advent of KMO crystal structures as researchers began to model potential inhibitors to that of known inhibitors and native substrates of KMO (Figure 5). *m*-Nitrobenzoyl alanine (*m*-NBA), shown to have an IC50 of 900 nM against KMO from resuspended, homogenized rat organs, was the first published specific inhibitor for KMO. In addition to its inhibitory activity, levels of the toxic metabolite 3-HK were decreased, and neuroprotective KYNA levels increased in rodents dosed with 400 mg/kg of m-NBA [123]. Improving on the structural aspects of substrates, halogen substitution on the aromatic rings at the third and fourth positions (3,4 dichloro and 3, 4 difluoro derivatives) were shown to produce more potent inhibitors [124]. Soon 3,4-dichlorobenzoyl alanine (3, 4-CBA or FCE 28833) (Figure 7) was developed showing similar, but more potent, benefits to m-NBA. This compound, shown to have an IC50 of 200 nM against KMO, became a favorable contender to be used therapeutically after it was shown that it could inhibit the synthesis of quinolinic acid by preventing interferon gamma in primary cultures of human monocyte-derived macrophages [125]. It was soon discovered that while substrate analogues bind in the active site of KMO and inhibit activity, they also detrimentally result in the formation of cytotoxic hydrogen peroxide by uncoupling the reaction of NAD(P)H and O_2_ [112]. Because of this, the development of KMO inhibitors using substrate analogues was abandoned.

**Figure 8:**
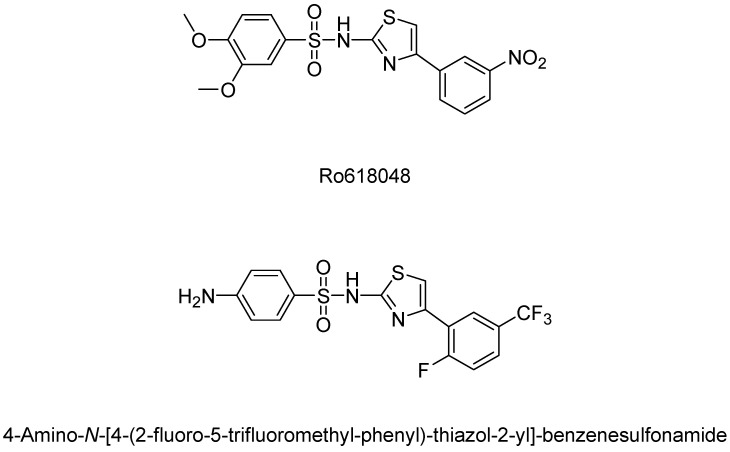
KMO inhibitors developed by Roche Pharmaceuticals.

After the failed attempt to model KMO inhibitors after native substrates, scientists began to move towards research on more novel and complex compounds. The most profitable idea came from researchers at Roche Pharmaceuticals when they decided to develop derivatives from the substrate analogue m-NBA. Their modeling led to the development of potent KMO inhibitors, N-(4-phenylthiazol-2-yl)benzenesulphonamide derivatives. In this series of compounds, sulfonamides replace the carboxylic acid moiety to not only improve potency, but also allow the compound the ability to pass through the blood–brain barrier. Sulfonamides are attractive because they reduce the polar surface area and have greater log P profiles, which markedly improve the blood–brain barrier permeability. These derivatives yielded the most potent KMO inhibitors at that time and produced the most well-known KMO inhibitor 3,4-dimethoxy-N-[4-(3-nitrophenyl)thiazol-2- yl]benzenesulfonamide (Ro-61-8048) with an IC50 of 37 nM and 4-amino-N-[4-[2-fluoro-5-(trifluoromethyl)phenyl]- thiazol-2-yl]benzenesulfonamide having an IC50 of 19 nM (Figure 8) [126]. The potency of these compounds did not end in vitro, but continued in vivo as well. After orally administering these compounds to gerbils, it was shown that they inhibited KMO with ED50s in the range of 3–5 μmol/kg in gerbil brains. Not only did Ro-61-8048 show a greater potency than the previously discussed native substrate analogue 3,4-dichlorobenzoyl alanine, but the dosing of Ro-61-8048 at 42 mg/kg orally also increased the concentration of KYNA seven-fold in the hippocampus of rats.

UPF648, chemically known as 2-(3, 4-dichlorobenzoyl)-cyclopropane-1- carboxylic acid, is another extensively studied inhibitor of KMO. Previously in this chapter, it was discussed that UPF648 prevents the binding of the native substrate KYN by binding closely to the FAD cofactor [115]. UPF648 has been shown to shift the kynurenine pathway towards a state of neuroprotection in rodent models when peripherally administered by diminishing the concentrations of neurotoxins 3-HK and QUIN and giving rise to the neuroprotective metabolite KYNA [127]. In a transgenic *Drosophila melanogaster* model of Huntington’s disease, UPF648 was shown to mitigate disease-relevant phenotypes, providing the first genetic evidence in animal models that KMO inhibition can sequentially lead to protection against neurodegenerative diseases and other disorders that result from the overactive stimulation of the kynurenine pathway. After hearing information about the inhibitory effects of UPF-648, one would think it to be an ideal drug for KMO inhibition. It was found, however, that while UPF648 inhibits KMO, it also significantly increases the production of hydrogen peroxide by almost 20-fold. In its absence 0.0032 μM/min of H_2_O_2_ is produced. In the presence of UPF 648, H_2_O_2_ production increases to 0.056 μM/min, indicating some destabilization of the flavin intermediate formed via the natural catalytic cycle of flavin containing monooxygenases. It also, unfortunately, does not cross the blood–brain barrier [115]. Nevertheless, UPF648 serves as an important model in developing KMO inhibitors as it has been revealed that the binding architecture of yKMO with UPF648 is more or less identical to human KMO.

**Figure 9:**
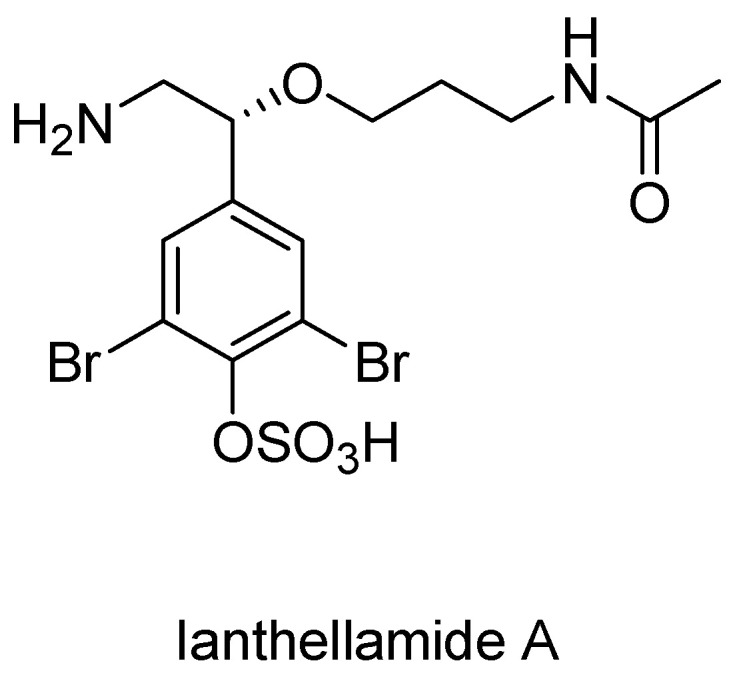
Ianthellamide A, a selective kynurenine-3-hydroxylase inhibitor from the Australian marine sponge Ianthella quadrangulate.

Recently the discovery of a novel octopamine derivative isolated from the Australian marine sponge Ianthella quadrangulata, Ianthellamide A, has presented another lead structure (Figure 9). In 2012, Ianthellamide A was shown to selectively inhibit KMO with an IC50 of 1.5 μM [128]. Structurally, it is obvious that this compound differs from the native substrate L-Kyn, revolutionizing the understanding of the rigid binding pattern of substrates in the KMO active site. It remains to be seen, however, whether Ianthellamide A produces adequate levels of KYNA in the brain to serve as a neuroprotectant. If future animal models prove to demonstrate neuroprotective effects, Ianthellamide A will not only be used to model novel KMO inhibitors, but could also possibly serve as therapy for the treatment of neurodegenerative diseases.

With the exception of Ianthellamide A, all of the inhibitors mentioned above prove the innovative effects the rational structure-based design has on drug development. As mentioned, several studies have determined KMO inhibition to raise KYNA levels leading to neuroprotection. In order to further advance the discovery of a KMO inhibitor, scientists must find a compound that can penetrate the blood–brain barrier and does not stimulate FAD reduction by NADPH. The pharmacological features of the inhibitors described above should allow a more detailed investigation of the role of the kynurenine pathway in certain neurological disorders, in particular those associated with neuroinflammation.

## 4. Pharmacophore Development

Two main strategies are used with pharmacophore modeling depending on the availability of the bound conformation of a ligand with its target receptor. Lacking ligand-target structural data, model building involves the alignment of the proposed binding features of a series of active molecules (i.e., the recognition of patterns common to the active compounds but lacking in similar compounds that are not active). The common features of a series of active compounds ultimately represent a pharmacophore model, and, in a typical research project, hundreds of models are generated and prioritized using internal scoring tools, as well as external scoring methods such as GH-scoring [129,130]. In the ligand-based approach, the proposed models must also consider stereoisomerization, tautomerization, ionization states, and conformational flexibility. Such complexities can be addresses during the database construction or within the pharmacophore definition. Many modern systems, such as Catalyst [131] and Phase [132], address stereoisomerization and ionization states as part of the database construction. Conformational flexibility can also be addressed through database construction (by registering multiple diverse conformations) or via using sophisticated querying techniques such as flexible queries, [133,134] or both.

Receptor-based pharmacophore modeling, however, eliminates many of the complexities of model development, described above, since a bound conformation of an active ligand is already available. Using the bound conformation, a series of pharmacophore models can be generated by the selective use of the chemical features of the compound in its active (i.e., bound) conformation. A good example of the success of receptor-based pharmacophore modeling can be found in [135].

### 4.1. KMO Inhibitor Pharmacophore Development

**Figure 10:**
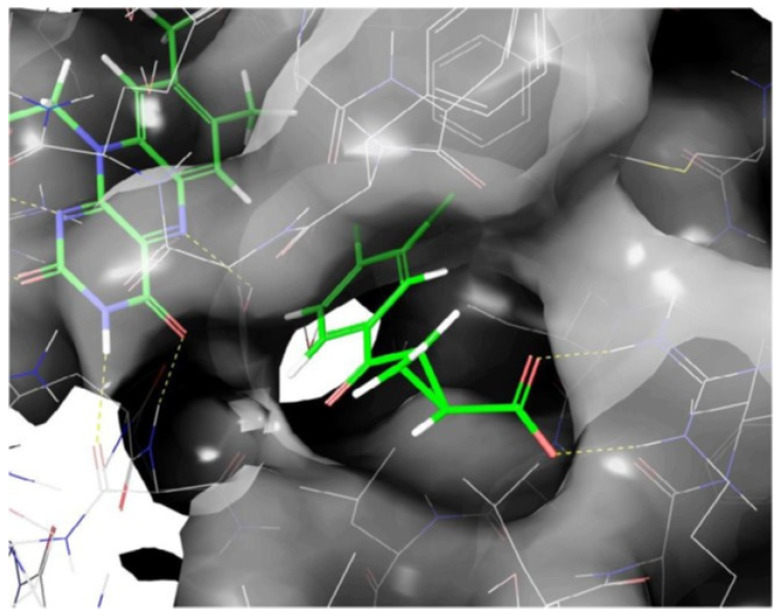
The X-ray crystal structure complex of UPF648 bound to yeast KMO, reprinted with permission from Ref. [136].

Receptor-based pharmacophore modeling approaches were used in our research to discover novel KMO inhibitors. Figure 10 displays the X-ray crystal structure complex of the active ligand, UPF648, bound to yeast KMO (PDB code: 4J36). This X-ray structure was the basis for the pharmacophore modeling.

**Figure 11:**
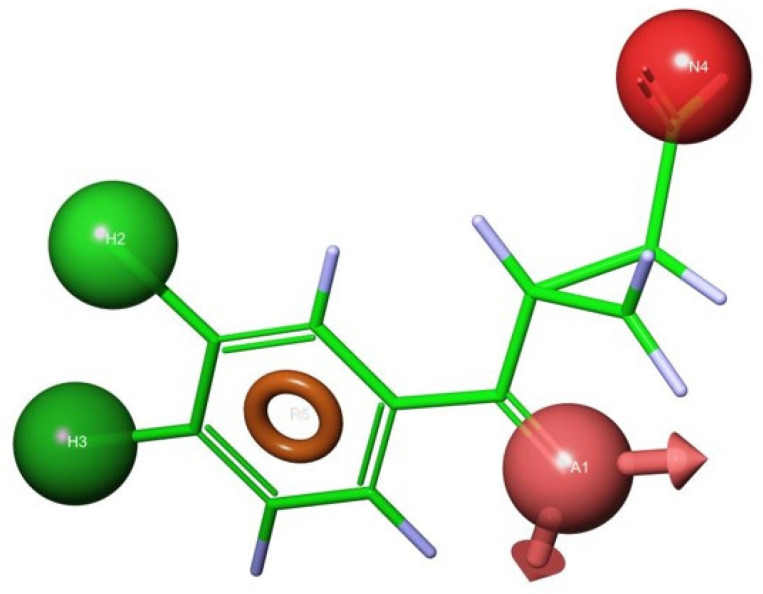
Selection of features to constitute the pharmacophoric centers. An aromatic center (orange), H-bond acceptor (pink), acidic group (red), and two lipophilic moieties (green).

**Figure 12:**
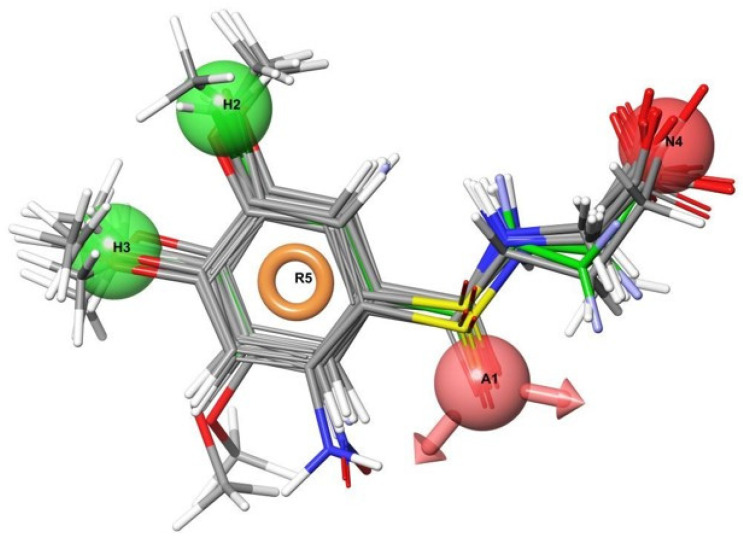
A number of compounds retrieved from the commercially available Sigma-Aldrich database.

The in silico extraction of the ligand in its bound conformation provided the foundation for the pharmacophore models. Four critical pharmacophoric centers were identified: (a) two hydrophobic centers, (b) an aromatic ring, (c) an electron rich hydrogen bond acceptor group, and (d) an acidic group. Figure 11 displays the UPF648, in its bound conformation, with the pharmacophoric centers highlighted. Once the pharmacophoric centers are divorced from the original structure, it can be used for database searching. A number of compounds was retrieved from the commercially available Sigma-Aldrich database Figure 12.

**Figure 13:**
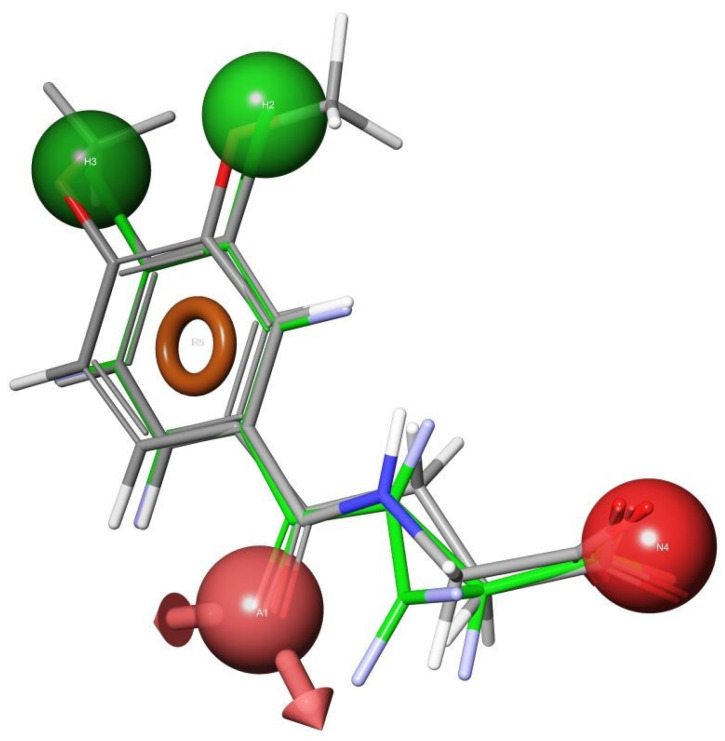
Two compounds aligned with UPF648 (green bonds) tested for biological activity reprinted with permission from Ref. [136].

Two compounds of our top scoring compounds were aligned with UPF648 (Figure 13). The compounds were tested for inhibition, and one of the two, 3,4-dimethoxyhippuric acid, had a K_i_ of 33 µM [136]. The discovery of KMO inhibitors based on the computational work demonstrated the proof of concept. The pharmacophore model is being expanded and used for ongoing and future research.

### 4.2. Inhibition Studies

KMO is an NADPH-dependent flavoenzyme. The first step in the catalytic mechanism of KMO involves the binding of L-Kyn/ligand to the KMO–FAD complex; next, NADPH binds to the complex to reduce the FAD and leaves as NADP^+^. This step is followed by the binding of molecular oxygen to the ligand–KMO–FAD complex to form a hydroperoxide intermediate, followed by the product release and water molecule [112]. The binding of a ligand to the KMO–FAD complex causes perturbance in the FAD fluorescence until the active site is fully saturated. Therefore, the change in FAD fluorescence at the wavelength of 520 nm (FAD emission) can be used to measure the dissociation constant (K_d_). This method was introduced and thoroughly described by Moran et al. [62].

On the other hand, though kynurenine analogs have shown a very appreciable inhibitory potency towards KMO, many have been reported to cause NADPH uncoupling [112,120], which leads to the accumulation of hydrogen peroxide, a less desired side effect of the compounds. Therefore, to confirm that a proposed KMO inhibitor, especially a substrate analog, indeed inhibits KMO without uncoupling NADPH, a UV–Vis spectrophotometer can be used to measure the decrease of NADPH absorbance at 340 nm. The lack of a high blank rate in the presence of an inhibitor during this measurement suggests that the ligand binds to KMO not as a substrate or an NADPH uncoupler, but as a competitive inhibitor.

However, when a high blank rate is observed, a HPLC analysis can provide further insights on the binding mode of the ligand. If the ligand (substrate analog) is a KMO inhibitor, then, there is an increasing of absorbance in the 370–400 nm region, indicating that the hydroxylation took place. Non-substrate effectors of KMO do not cause this change in absorbance.

## 5. Discussion and Conclusions

The kynurenine pathway is the major pathway for tryptophan metabolism. Recently, this pathway has gained much attraction in research due to its metabolites—metabolites that either lead to protection or destruction in the body. Of major interest are the metabolites stemming for KMO, the pivotal enzyme of the kynurenine pathway. KMO activation can either lead to an accumulation of toxic products or produce kynurenic acid to counteract this toxicity. Regarding drug design and discovery, the kynurenine pathway is an attractive target for the development of inhibitors, and the modulation of KMO presents a major point of interest for pharmacological investigations on mechanisms that either shift the KP towards glutamatergic agonism (QUIN) or antagonism (KYNA)—generally, a shift towards a KYNA product is preferred. The over-activation of KMO has been implicated in numerous pathologies, and the inhibition of this enzyme produced promising therapeutic results. The goal of our research was to focus on the design, synthesis, and biological testing of novel inhibitors that may serve as lead compounds to reduce unwanted side effects and toxicity observed in KMO upregulation. These new drug entities target the kynurenine pathway of tryptophan metabolism in humans, specifically kynurenine monooxygenase (KMO). Drugs that can block the formation of kynurenines and/or quinolinate by inhibiting one or more of the kynurenine pathway enzymes could improve the prognosis of patients with many diseases described in this review article.

Future research should focus on the identification of compounds that efficiently bind to KMO and may be considered as good candidates for future development into potent inhibitors. This review lays the foundation for future research investigating how small molecules interact within the KMO binding pocket. Several areas in which this research can be utilized has been envisioned.

Though the computational portion of this project is nearly complete, several avenues in the production of novel KMO inhibitors remain to be explored. Logically, the next step for this structure-based design project is to generate co-crystal structures of each compound and study their enzymatic activity. The co-crystallization of each compound will assist in validating the information presented in the thesis. Additionally, our synthetic chemistry collaborators can study the observed interactions in each structure and create more potent binding agents that could feed back into modeling and be used for refining KMO inhibitors. In future iterations of molecular docking of compounds, the development of rules to select ligands that may bind to the KMO enzyme with affinity and specificity may be necessary. From these studies, the value of virtual screening as a precursor to the full synthesis of KMO inhibitors may be revealed.

Using hKMO for inhibition studies and crystallographic studies is ideal. However, attempts for its purification using recombinant protein technology remain unsuccessful [137]. Given that hKMO is a membrane protein, a detergent is required for its purification, which is detrimental for a successful crystallography [138]. Therefore, pfKMO is often seen and used as a perfect model for studying KMO inhibition that would translate in drug discovery. However, some non-substrate analogs such as Ro 61-8048 and UPF648 have been shown to have distinct binding modes in various KMO species [120]. For instance, Ro 61-8048 is a competitive inhibitor of scKMO, whereas it acts as a non-substrate effector in both hKMO and pfKMO where it triggers the accumulation of hydrogen peroxides. Therefore, future studies should aim to determine not only great leads and their respective binding modes, but also a better structure as a surrogate of hKMO.

## Data Availability

Data is available upon request of the authors.
